# Vestibular granuloma as a rare post-stapedotomy complication: case report and literature review

**DOI:** 10.1007/s00405-025-09880-1

**Published:** 2025-11-25

**Authors:** Riccardo De Lellis, Filippo Valentini, Mara Riminucci, Alessandro Corsi, Francesca Yoshie Russo, Daniele De Seta

**Affiliations:** 1 Department of Sense Organs, Sapienza University of Rome, Viale del Policlinico 155, Rome, 00161 Italy; 2 Department of Molecular Medicine, Section of Pathology, Sapienza University of Rome, Viale del Policlinico 155, Rome, 00161 Italy; 3 Unit of Otolaryngology Head and Neck Surgery, Department of Sense Organs, Policlinico Umberto I – Sapienza University of Rome, Viale del Policlinico 155, Rome, 00161 Italy

**Keywords:** Granuloma, Otosclerosis, Stapedotomy, Complication, Stapes-prosthesis, Vestibular disorders, PPPD

## Abstract

**Background:**

Vestibular granuloma is a rare complication of a common surgical procedure, such as stapedotomy; it can significantly impact the outcome of surgery, leading to severe impairment and deterioration in the patient’s quality of life.

**Case report:**

A 59-year-old male underwent stapedotomy with a titanium prosthesis. Initially, hearing function improved but, within days, the patient developed vertigo and worsened hearing loss. HR CT of temporal bone revealed soft tissue in the tympanic cavity, prompting revision surgery. Granulomatous tissue surrounding the prosthesis was removed, leading to symptom relief. However, the patient later developed Persistent Postural-Perceptual Dizziness (PPPD), requiring SSRI therapy.

**Discussion:**

The sudden onset of persistent vertigo and hearing loss, that rapidly worsens in post-operative setting, represents a crucial red flag for the surgical team. Hearing assessments and imaging play a key role in quickly diagnosing the cause of these complications, which may be attributed to an inflammatory response at the surgical site, possibly due to granuloma formation. This enables the surgical équipe to swiftly implement appropriate care strategies for the patient, even in the presence of additional and subsequent complications like PPPD, which requires a comprehensive, multimodal approach to treatment.

**Conclusion:**

This case highlights the risk of a series of complications after surgery and even after reoperation, ultimately requiring conservative multimodal treatment and follow-up. Early recognition and coordinated care are essential for optimal long-term management of patients, even in the presence of unfavorable outcomes and complications.

## Introduction

Despite its high-success rate, the stapedotomy – as a widely performed surgical procedure – is not exempt from complications, some of which can significantly affect its post-operative outcomes. One such complication is the development of vestibular granuloma, which is a devasting, rare and clinically relevant inflammatory reaction that can occur in the inner ear in the early postoperative period. The first signs of this complication affect the patient’s hearing and vestibular function, both of which progressively and rapidly deteriorate in the post-operative period; this decline becomes even more insidious and unexpected after a stapedotomy that appeared to have been successful.

Vestibular granuloma, also known as “reparative granuloma”, is characterized by an exaggerated tissue response and it was first described by Kaufman and Schuknecht [1] as a pathologically augmented normal reparative process, which lead to this rare after-surgery complication, occurring in 0.07% of stapedotomy to 0.1% of stapedectomy cases [2].

Vestibular granuloma is likely triggered by surgical trauma, foreign body reaction, or inflammatory mediators. Clinically, it presents with a well-established symptomatology such as vertigo, imbalance, tinnitus, and hearing loss up to profound deafness, which can mimic other more common and milder post-stapedotomy complications, making its early diagnosis challenging [3]. The severity of the symptoms and their rapid worsening should raise suspicion – within a reasonable time frame – of the development of such complication, so that the proper diagnostic and therapeutic process may be promptly initiated.

Even though therapeutic management is not yet well-standardized, current treatment approaches range from conservative medical therapy to revision surgery, depending on the severity of symptoms and the extent of the lesion [2].

Our article aims to review the current understanding of vestibular granuloma following stapedotomy. Concurrently, we bring our experience with a clinical case of granuloma of the vestibule following stapedotomy surgery, hoping to contribute to the improvement of patient outcomes and surgical decision-making in otologic practice.

## Case report

A 59-years-old male patient presented with clinical signs of left otosclerosis, a progressive left side conductive hearing loss, with a type-A-tympanogram and absence of ipsi- and contralateral stapedial reflexes. The first pre-operative temporal bone CT scan showed no signs of otosclerosis but ruled out any other fixation of the ossicular chain. The patient was unable to undergo a magnetic resonance imaging (MRI) scan for further diagnosis and in-depth analysis due to cardiac problems.

Thus, the patient underwent surgery under local anesthesia in one-day-surgery setting. Intraoperatively, the fixation of the stapes and motility of the incus and malleus was confirmed, microdrill calibrated platinotomy and removal of stapes suprastructure was performed and a calibrated 0.5 mm-diameter 4.75 mm-length titanium stapes prosthesis was positioned. The surgery was uneventful, and the patient reported immediate post-operative improvement of hearing function and was discharged the following day with antibiotic ear drops. Three days after, the patient reported vertigo and oral corticosteroid treatment was prescribed.

**Fig. 1:**
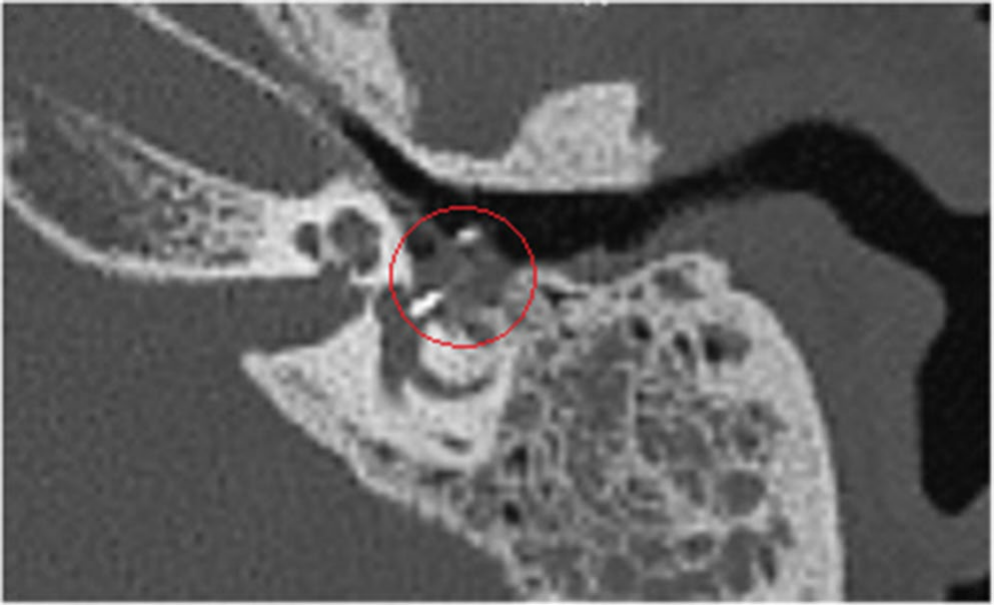
Left-ear and mastoid multiplanar reconstruction CT scan (post-operative, first surgery), showing material of soft tissue consistency around the prosthesis and in the middle ear (circled)

One week after surgery, at the check-up visit, Weber test lateralized to the left ear, with persisting vertigo and tinnitus. The patient continued corticosteroid therapy, prolonging its initially planned duration. The audiometric examination, performed two weeks after surgery, revealed a severe left mixed-form hearing loss. The control CT scan performed one month after surgery revealed soft tissue density material occupying the left tympanic cavity, stapedial prosthesis was correctly in place and well-articulated with the ossicular chain, prosthesis’ piston was likewise in place, with intra-vestibular penetration of 0.8 mm (Fig. 1).

A further follow-up audiometric examination showed a severe left unilateral sensorineural hearing loss.

**Fig. 2:**
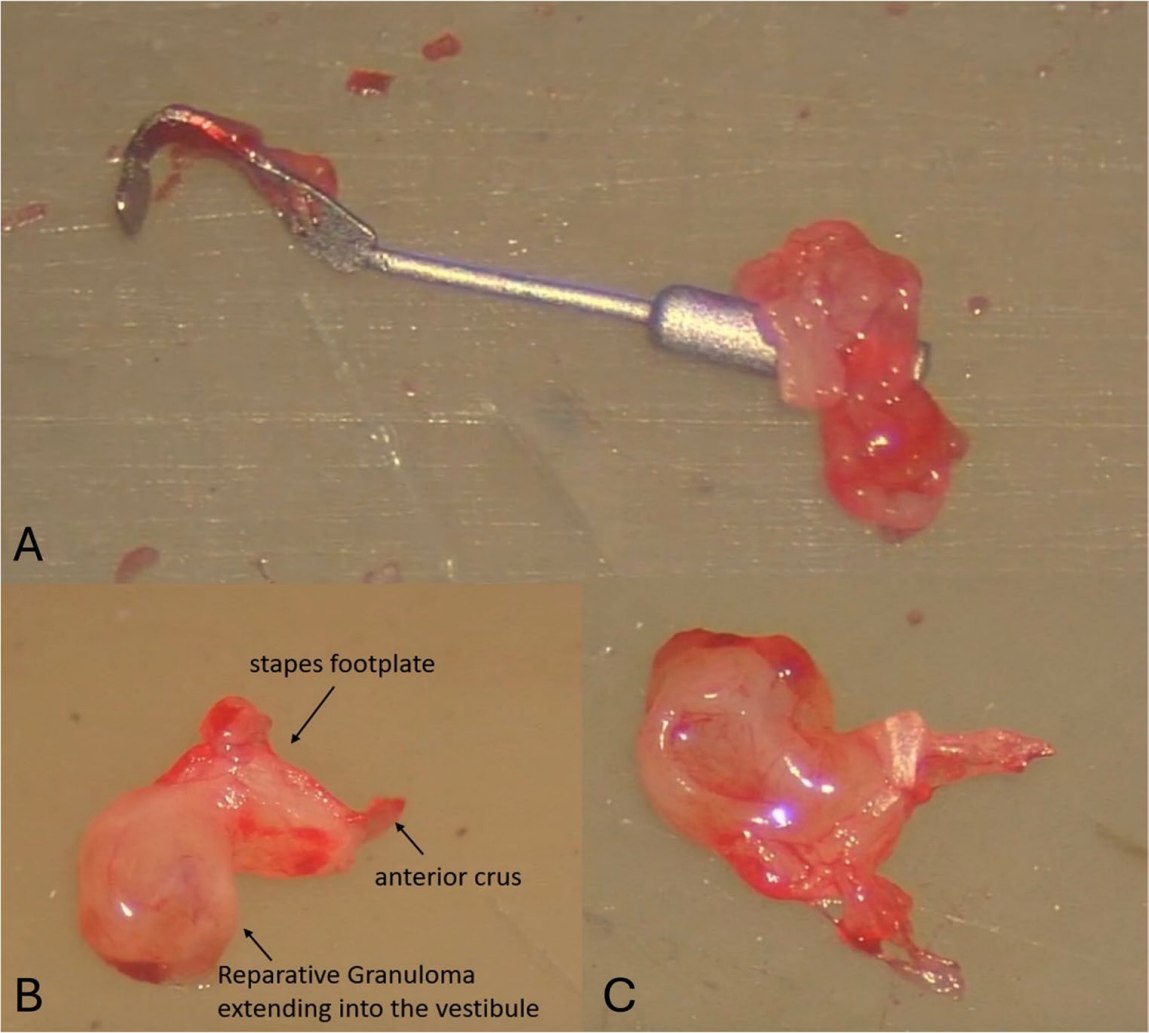
The stapedial prosthesis (A) surrounded by the harvested tissue from left middle ear, intraoperative during revision surgery (B, C)

Revision surgery was performed less than two months after the initial procedure. Intraoperatively, the tissue occupying the middle ear, surrounding the stapedial prosthesis, was removed with the prosthesis as well **(**Fig. 2A**)**. The platina was removed with the mass of tissue extending intravestibularly from the platinal plane (Fig. 2B, C). Perichondrium was taken from the tragus, and the oval window was sealed.

**Fig. 3:**
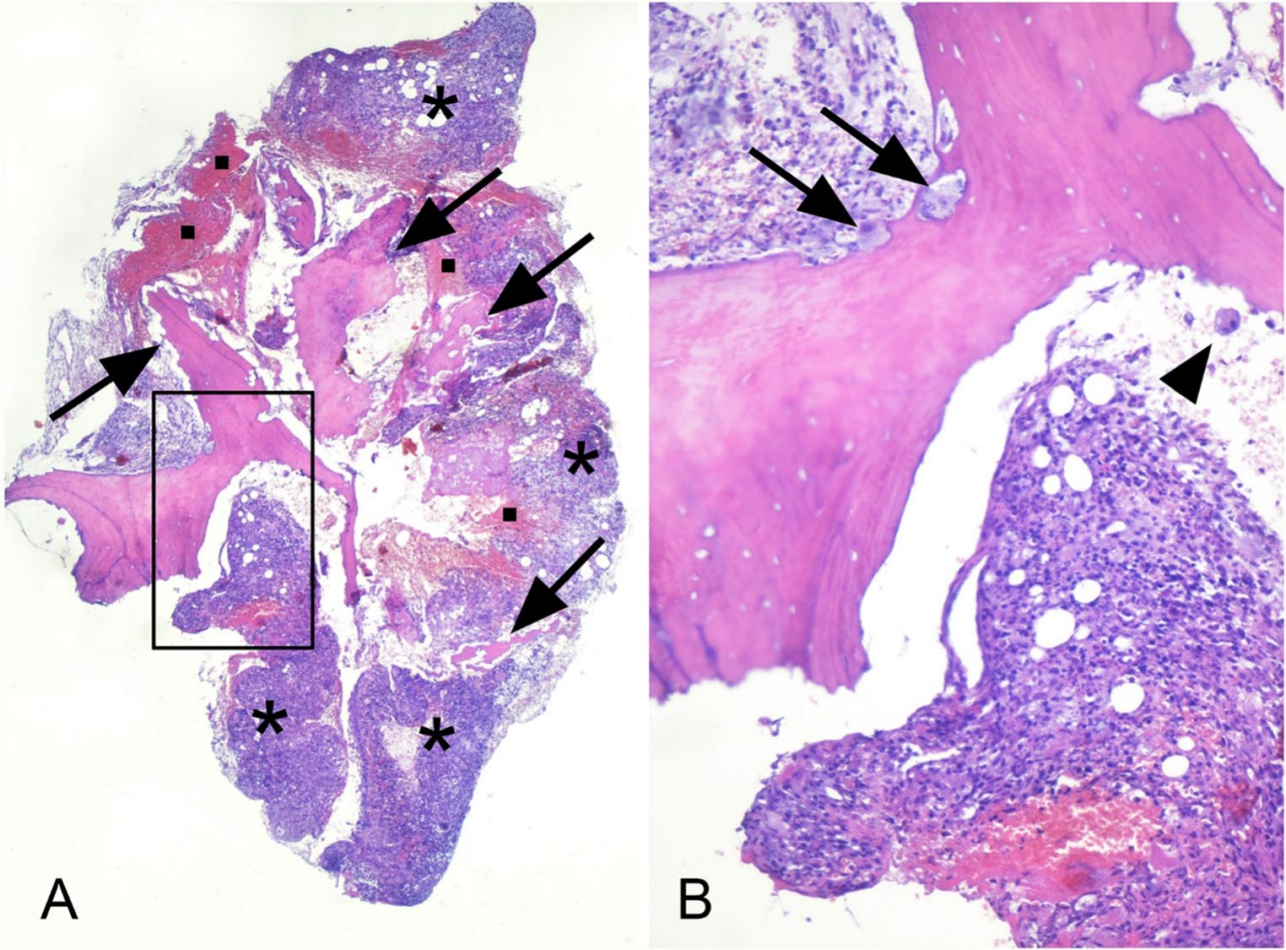
Low- and high-power magnifications of the excised tissue are shown in A and B respectively. The box in panel A illustrates the area shown in panel B. Arrows, asterisks and squares in panel A identifies the fragments of bone trabeculae, the inflammatory cells and the hemorrhages respectively. In the portion of the bone trabecula illustrated in B osteocyte lacunae are empty, as for osteonecrosis, and osteoclasts, in contact (arrows) or not (arrowhead) with its surface, are abundant. A and B: hematoxylin-eosin

Histological examination of the excised sample revealed fragments of bone trabeculae with empty osteocyte lacunae, as for osteonecrosis, and with extensively scalloped surfaces associated with inflammation and hemorrhages (Fig. 3A, B). Osteoclasts, either in contact with or detached from the bone surfaces, were abundant (Fig. 3B) as the result of inflammation-dependent activation of bone resorption.

**Fig. 4:**
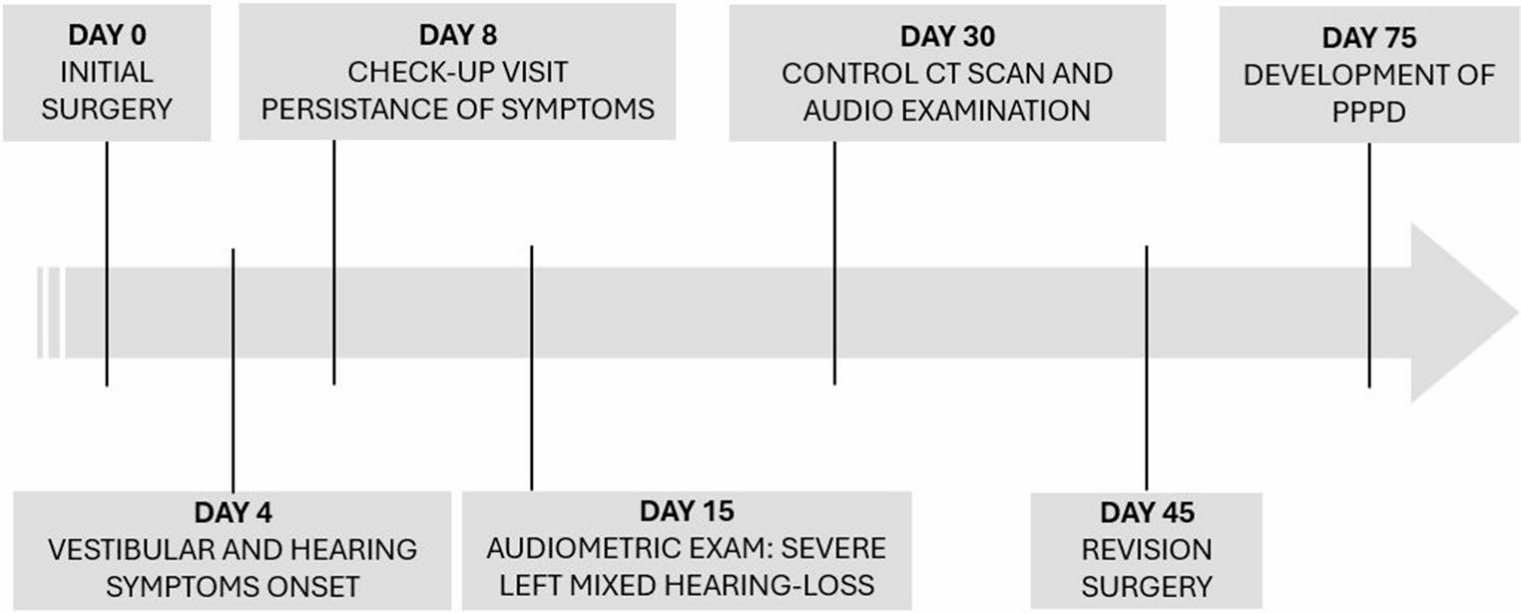
Visual timeline summarizing key chronological events

In the immediate postoperative phase, the patient experienced marked improvement in dizziness, hearing and imbalance symptoms. One month after revision surgery, the patient developed Persistent Postural-Perceptual Dizziness (PPPD) and SSRI oral therapy was prescribed. In the timeline shown in Fig. 4, the key events are visually and chronologically described.

Although MRI would have been useful for studying the local clinical case, it was not performed due to patient’s cardiological contraindications. Therefore, the patient’s subsequent follow-up was instrumental, with CT scans, and clinical at the vestibular disorders center of the hospital ENT department.

## Discussion

Surgical materials, including those used in stapedotomy, are known to be inert and biocompatible and therefore unlikely able to trigger foreign body reactions, that can lead to persistent inflammation and granuloma formation. The inflammatory cascade primed by the immune system initiates a macrophage activation, giant cell formation, and fibroblast proliferation and this response can result in an excessive granulation tissue formation, which may extend into the vestibule, interfering with normal labyrinthine function [4].

The sustained inflammatory milieu within the middle and inner ear, possibly driven in part by autoimmune mechanisms, contributes to the worsening of postoperative outcomes [5]. While materials such as hemostatic sponge are designed to be resorbed over time, incomplete absorption or an excessive inflammatory response may lead to prolonged tissue irritation and to the formation of vestibular granulomas. In this sense, it is worth noting that in our clinical case, the hemostatic sponge was only placed in the external left ear canal to stabilize the tympano-meatal flap, and nothing was placed in the oval fossa.

Some authors argue that certain materials used during surgery, but not intended to stay in place, such as cotton wool, linen fibers from patches, talc or starch powder from gloves, fragments of Teflon, may lead to irritative-inflammatory responses in the tympanic cavity with the subsequent formation of the oval-window granuloma [6], while Kaufman et al. [1] and Burtner et al. [7] claim the opposite.

Foreign body reactions may also be triggered by the materials used in the construction of the prostheses themselves, such as Teflon (polytetrafluoroethylene, PTFE), platinum, titanium, fluoroplastic materials, ceramic and hydroxyapatite [8]. While these materials are biocompatible, some patients may develop a delayed hypersensitivity reaction to the implanted device. Among these mentioned, titanium prostheses have proven to be excellently compatible in a large case series, more than prostheses made of other materials that are burdened by a higher rate of recurrences, even across different surgical techniques of prosthesis implantation [9].

Available evidence in the literature suggests vestibular granuloma can frequently occur in association with the objective finding of prosthesis malposition, evident both on control CT images and in further exploratory tympanotomy or intraoperatively at the time of revision surgery, thus leading to substantial surgical failure ab initio [10]. In our case, CT imaging confirmed the correct positioning of the stapedial prosthesis, even if massively affected by the inflammatory granuloma.

Clinically, patients who develop an inflammatory vestibular granuloma may report a severe hearing loss on the affected side, tinnitus, vertigo and imbalance with serious difficulty standing and walking properly. In some cases, this symptom complex can be recurrent or progressively worsening, early-onset or delayed, and it may occur all at once or may appear with different manifestations at different times. These warning signs should prompt the surgeon to implement a treatment approach for the patient as soon as possible.

A recent retrospective study highlighted the central role of revision surgery in patients who develop a granuloma of the vestibule. Antibiotic and corticosteroid therapy provides an unavoidable consolidation of the outcome and a maintenance of the results obtained with surgical revision [11]. On the other hand, subsequently, the management of PPPD is not yet standardized and is currently based on a multidisciplinary approach involving vestibular rehabilitation, pharmacotherapy, and psychological counseling [12].

At present, there is no direct causal link – clearly documented – between the presence of a vestibular granuloma and the development of PPPD, defined as a prolonged, persistent and relapsing dizziness, a sense of unsteadiness and imbalance, which often recur daily and seriously affects the life and daily activities of the affected patient [13]. This would suggest that this condition is not caused by the granuloma itself, but rather derives indirectly from a functional insult to the vestibular and therefore labyrinthine structures of the inner ear, ipsilateral to the lesion.

This case report is affected by various certain limitations. First, the findings are based on a single patient, which limits the generalizability of the results to a broader population, even though the reported experience remains relevant and nonetheless applicable to other patients who fall into a similar case history. Due to the observational nature of the report, causal relationships cannot be definitively established. Furthermore, the lack of postoperative MRI due to the impossibility of performing them because of the patient’s cardiac problems, limited our ability to correctly evaluate the intra-vestibular spaces and the possible presence of a granuloma.

## Conclusions

The case we are discussing represents a rare and dramatic occurrence of post-operative granuloma following stapedotomy with a titanium prosthesis, leading to a profound sensorineural hearing loss followed by the development of PPPD after the revision surgery. It is relevant to emphasize that the prostheses currently used in stapes surgery, particularly those made of titanium or other few materials, are widely employed and considered safe due to their excellent biocompatibility and the very low incidence of complications.

Despite the rarity of such adverse events, in routine surgical procedures such stapedotomies, our experience highlights the unavoidable need for careful post-operative follow-up and evaluation, even more in patients who develop atypical or persistent symptoms after surgery. Proper information and informed consent is essential in this as in all situations. The multidisciplinary approach, involving ENT specialists, audiologists, neurologists, psychiatrists, and vestibular rehabilitation specialists is crucial for optimal patient management and functional recovery, both following the development of vestibular granuloma and after the onset of PPPD. Additional evidence and case series with follow-up of similar patients are surely needed to provide supplemental data in supporting and establishing potential predisposing factors.

Further research is warranted to investigate the potential link between vestibular granuloma development following stapedotomy and underlying autoimmune conditions. Currently, there is no scientific literature specifically addressing this association. Given the role of dysregulated immune responses in chronic inflammation and aberrant tissue repair, it is crucial to elucidate whether autoimmune disorders predispose patients to this kind of complication. Future studies should focus on identifying specific immunological markers, assessing the impact of systemic autoimmune disease on wound healing in the inner ear, and evaluating potential preventive or therapeutic strategies. A better understanding of these mechanisms could lead to personalized risk assessment, improved perioperative management, prevention strategies, and targeted interventions to optimize surgical outcomes in affected patients.

## Abbreviations

CT: Computed Tomography
MRI: Magnetic Resonance Imaging
ENT: Ear-Nose and Throat
SSRI: Serotonin Reuptake Inhibitors
PPPD: Persistent Postural-Perceptual Dizziness
